# The reciprocal relations between experiential avoidance, school stressor, and psychological stress response among Japanese adolescents

**DOI:** 10.1371/journal.pone.0188368

**Published:** 2017-11-22

**Authors:** Kenichiro Ishizu, Yoshiyuki Shimoda, Tomu Ohtsuki

**Affiliations:** 1 University of Toyama, Toyama, Japan; 2 Saga University, Saga, Japan; 3 Waseda University, Saitama, Japan; University of Cyprus, CYPRUS

## Abstract

The present study aimed to investigate the reciprocal relations between experiential avoidance, stressor, and psychological stress response (which consist of anger, depression, anxiety, helplessness, and physical complaints). In this study, 688 Japanese junior high school students (353 boys, 334 girls, 1 unidentified; mean age 13.28 years) completed three waves of questionnaires on experiential avoidance, stressor, and psychological stress response, with one-week intervals between measurement waves. Results from cross-lagged panel analyses showed that experiential avoidance predicted subsequent stressor and psychological stress response. Furthermore, the stressor and psychological stress response influenced by prior experiential avoidance affected subsequent occurrence of experiential avoidance. The findings suggest that reciprocal relations exist among the variables, and that the interaction between experiential avoidance and psychological stress was possible in adolescents.

## Introduction

Acceptance & Commitment Therapy (ACT) is a psychotherapeutic approach that has philosophical and theoretical bases in functional contextualism and Relational Frame Theory (RFT), respectively. ACT has been applied to a wide variety of mental illnesses, and has shown to be effective for chronic pain, depression, anxiety disorders, psychotic symptoms, drug abuse, and other conditions [1, 2].

Experiential avoidance (EA) is a key concept used to explain and predict a wide variety of mental health and behavioral issues during ACT. EA refers to maladaptive coping styles and attitudes that aim to avoid negative thoughts, emotions, and sensations [3]. It defines a functional class of behaviors that occur when a person is unwilling to remain in contact with uncomfortable private experiences, such as bodily sensations, emotions, thoughts, and memories [4]. EA can be a problematic behavior because private experiences are hard to change, or are even paradoxically strengthened by control efforts [5]. Thus, previous studies have suggested that EA is likely to be associated with psychopathology across the broad spectrum, and with a lower quality of life [3, 6].

There is a growing body of evidence demonstrating that EA is associated with a variety of mental and behavioral problems in adult samples. A meta-analysis by Hayes et al. [7] showed that EA generally had a moderate relation with mental health and behavioral issues. Spinhoven et al. [6] stated that EA might be a relevant transdiagnostic factor affecting the course and development of emotional disorders.

Although ACT is beginning to be applied to a wide range of adult clinical populations, the practice and basic research into ACT with children and adolescents have just recently begun to be explored. The Avoidance and Fusion Questionnaire for Youth (AFQ-Y) is a well-established child-report measure of EA [8], and subsequent studies on adolescent EA have been conducted using this scale. Previous studies have also provided evidence that EA is associated with variables related to mental health in adolescents, such as depression [9, 10,11], anxiety [11, 12], self-injury [13], alexithymia [14], borderline tendency [15, 16], and well-being [11, 17]. Hence, EA may develop and maintain psychological maladaptation, or the internalization versus externalization of problems.

Psychological stress has been linked to a wide array of psychological and physiological pathologies. Bardeen & Fergus [18] revealed that both cognitive fusion and EA exhibited significant positive associations with indices of emotional distress (i.e., anxiety, depression, and posttraumatic stress). EA is thought to play an important role in the development of psychological stress responses following stressful life experiences [19]. Though stress responses, including depression and anxiety, are a common problem among adolescents [20], the link between EA and psychological stress response is currently under-developed. Therefore, it is important to investigate whether EA plays a role in the occurrence or the *process* of psychological stress response.

There has been some effort to explore the prospective relation between EA and mental health in adolescents. For example, Sharp et al [16] showed that EA predicted subsequent borderline personality symptoms, and Bigan et al [9] demonstrated that EA was associated with high levels of depression using the latent growth model. Though research on EA and well-being among adolescents has been conducted for a while, few studies have examined the longitudinal relation between EA and psychological health in adolescents, and the process of EA in the maintenance of mental health remains insufficiently studied. Therefore, in this study, we aimed to investigate the relation between EA, daily stressful events (stressors), and psychological stress response via a short-term prospective design. We used a design with a very brief timeframe to investigate the temporal relation between EA and adolescent stress. In this study, we conducted a three-wave, three-week longitudinal study testing short-time reciprocal relation between EA, stressor, and psychological stress response to clarify the temporal relevancy among those variables.

## Materials and methods

### Participants and procedure

A total of 688 junior high school students in Japan (353 boys, 334 girls, and 1 unidentified, mean age = 13.28 years; SD = 0.66) completed the questionnaire. Before completing the questionnaire, participants were provided with oral and written explanations on the purpose of the study, their right to refuse to answer questions, and the protection of confidentiality. We also informed the participants’ parents via a printed document of our study purpose, their rights to refuse to answer, and our contact information. Students completed the questionnaire in their homeroom classes. All procedures were in accordance with the ethical standards of the responsible committee on human experimentation, and surveys were conducted under the Declaration of Helsinki. This study was approved by the Ethics Review Committee on Research with Human Subjects of Waseda University, Tokyo, Japan. This study was a short-term, 3-wave prospective study. As such, all participants were requested to complete the questionnaire three times, at one-week intervals.

### Measures

#### AFQ-Y (Avoidance and Fusion Questionnaire for Youth)

To measure experiential avoidance, we used the short version of the AFQ-Y for Japanese adolescents [21]. This measure is modeled after the Acceptance and Action Questionnaire (AAQ), which measures the same construct in adults. The original version of the AFQ-Y [8] is a 17-item, self-report measure designed to assess an aspect of psychological inflexibility engendered by cognitive fusion and experiential avoidance for adolescents. Previous research [8] revealed that the AFQ-Y had good internal consistency and convergent and construct validity. The Japanese version of the AFQ-Y was also found to have good validity by examining the relations among anxiety, depression, and suppression, and moderate internal and test-retest reliability [21]. In this study, we used the short form of the questionnaire, the AFQ-Y8 [8], for which the Cronbach’s alpha was .83. A higher score on the AFQ-Y8 indicates greater psychological inflexibility. Sample items include “My life won’t be good until I feel happy,” and “I am afraid of my feelings.”

#### Stressor and psychological stress response

The mental health checklist for junior high school students [22] was used to assess the stressor in school, as well as their psychological stress response. Participants responded to 12 items on stressor and 16 items on psychological stress response, and they were asked how often they experienced negative stressors in school per week (e.g., I could not get an expected result on an exam; I felt left out by my classmates). Psychological stress response involved measuring various stress-related symptoms including anger (e.g., I feel anger), depression/anxiety (e.g., I feel sad), helplessness (e.g., I can’t concentrate on my work), and physical complaints (e.g., I get tired easily). This checklist has been widely used to measure stress response in Japanese junior high school students; it has robust psychometric properties [22, 23], and overall scores could reflect the amount of perceived stress [24]. In the present study, Cronbach’s alphas were .81 and .92 for stressor in school and psychological stress response, respectively.

## Results

### Preliminary analyses

The means and standard deviations for all measures and the correlations between all the variables are presented in Table 1. As shown, significant positive relations between AFQ, stressor, and psychological stress response were found within-time and across-time, but the correlations were not high (*r*< .65), indicating that the threat of multicollinearity could be avoided.

**Table 1:** Means, Standard Deviations, and Correlations between all variables.

		Mean (SD)	Ⅰ	Ⅱ	Ⅲ	Ⅳ	Ⅴ	Ⅵ	Ⅶ	Ⅷ	Ⅸ	Ⅹ	Ⅺ
Ⅰ	T1 AFQ	8.12 (6.01)	-	.72**	.74**	.64**	.58**	.61**	.50**	.44**	.47**	.06	.03
Ⅱ	T2 AFQ	7.37 (6.28)		-	.74**	.49**	.62**	.56**	.38**	.49**	.46**	-	-
Ⅲ	T3 AFQ	7.25 (6.61)			-	.52**	.58**	.65**	.38**	.45**	.53**	-	-
Ⅳ	T1 Stress Response	9.60 (9.97)				-	.79**	.78**	.58**	.48**	.53**	.03	-.05
Ⅴ	T2 Stress Response	9.52 (10.31)					-	.84**	.46**	.58**	.55**	-	-
Ⅵ	T3 Stress Response	8.88 (10.54)						-	.48**	.50**	.59**	-	-
Ⅶ	T1 Stressor	8.23 (5.64)							-	.73**	.72**	.11**	.03
Ⅷ	T2 Stressor	7.54 (5.86)								-	.83**	-	-
Ⅸ	T3 Stressor	7.07 (6.22)										-	-
Ⅹ	age	13.28(0.66)										-	.01
Ⅺ	gender(boy = 0, girl = 1)											-	-

Note, AFQ = Avoidance and Fusion Questionnaire

** *p* < .01

### Cross-lagged panel model

To examine the unidirectional and reciprocal influence between AFQ, stressor, and psychological stress response across the three time points, cross-lagged panel analyses were conducted. The baseline model, including all within-time associations and auto regressive paths, showed a poor fit to the data (χ^2^ (21) = 232.151, p < .01; CFI = .942, GFI = .905, AGFI = .796, RMSEA = .147). Subsequently, we examined the model, including the reciprocal cross-lagged paths, which demonstrated a good fit to the data (χ^2^ (10) = 6.07, p = .809; CFI = 1.00, GFI = .997, AGFI = .987, RMSEA = .000). Compared to the baseline model with the model including the cross-lagged model [25], the delta chi-square was significantly changed (Δχ^2^ (11) = 226.08, p < .01); therefore, we adopted the latter model. As can be seen in Table 2, AFQ at T1 influenced psychological stress response and stressor at T2. In addition, psychological stress response and stressor at T2, in turn, predicted AFQ at T3. Moreover, AFQ at T2, which was affected by AFQ at T1, significantly affected psychological stress response and stressor at T3.

**Table 2:** Parameter estimates for the final cross-lagged model.

			Estimate		
Regression			standerdized	unstanderdized	SE
AFQ T1	→	AFQ T2	0.72**	0.77	0.03
		Stress Response T2	0.17**	0.30	0.06
		Stressor T2	0.10**	0.04	0.04
		AFQ T3	0.32**	0.37	0.05
		Stress Response T3	n.s	n.s	-
		Stressor T3	n.s	n.s	-
Stress Response T1	→	AFQ T2	n.s	n.s	-
		Stress Response T2	0.68**	0.69	0.03
		Stressor T2	n.s	n.s	-
		AFQ T3	n.s	n.s	-
		Stress Response T3	0.26**	0.27	0.04
		Stressors T3	n.s	n.s	
Stressor T1	→	AFQ T2	n.s	n.s	-
		Stress Response T2	n.s	n.s	-
		Stressor T2	0.67**	0.69	0.40
		AFQ T3	-0.09 *	-0.11	0.05
		Stress Response T3	n.s	n.s	-
		Stressor T3	0.18**	0.20	0.04
AFQ T2	→	AFQ T3	0.44**	0.46	0.04
		Stress Response T3	0.08**	0.13	0.05
		Stressor T3	0.08**	0.08	0.03
Stress Response T2	→	AFQ T3	0.14**	0.09	0.03
		Stress Response T3	0.62**	0.63	0.04
		Stressor T3	0.06 †	0.04	0.02
Stressor T2	→	AFQ T3	0.11**	0.13	0.05
		Stress Response T3	n.s	n.s	-
		Stressor T3	0.63**	0.69	0.04
Correlations			coefficient		
AFQ T1	↔	Stress Response T1	0.65**		3.16
AFQ T1	↔	Stressor T1	0.48**		1.60
Stress Response T1	↔	Stressor T1	0.57**		2.84
AFQ T2	↔	Stress Response T2	0.36**		1.29
AFQ T2	↔	Stressor T2	0.28**		0.80
Stress Response T2	↔	Stressor T2	0.40**		1.17
AFQ T3	↔	Stress Response T3	0.28**		0.90
AFQ T3	↔	Stressor T3	0.23**		0.78
Stress Response T3	↔	StressorsT3	0.36**		0.56

Note, AFQ = Avoidance and Fusion Questionnaire

*†* p < .10

* *p* < .05

**p < .01

## Discussion

In this study, we examined the temporal relations between EA, stressor, and psychological stress response using a three-wave, cross-lagged model.

First, we were able to confirm that EA influenced both psychological stress response and stressor, which suggests that, in a short time interval, EA contributes to the development of subsequent psychological stress response. This result was consistent with the statement by Hayes et al [4] that EA is associated with life distortion and with greater levels of psychopathology.

We also found that stressor and psychological stress response affected subsequent EA. This means that once EA evokes stressor and psychological stress response, these stressor and psychological stress response would provoke subsequent EA, which may indicate that EA, stressor, and stress response are related reciprocally within a short time. It has already been indicated that while EA would sometimes alleviate internal discomfort in the short term, avoidance of unwilling thoughts, emotions, and sensations actually increases the likelihood of experiencing them again in the future, thus elevating psychological distress and setting into motion a vicious cycle [16]. Plum et al [19] and Hayes et al [3] assumed EA was a factor that produces or increases psychological distress, but no empirical study has yet shown that EA is a factor affected by distress or stressor. It is important that, in this study, we showed empirically that EA is not only a factor that produces stressor and stress response but is also provoked by them as well.

Past research also examined the factors affecting EA. For example, Vanwoerden, et al [26] found that maternal disorganized attachment style predicted EA, and Moroz & Dunkly [27] demonstrated the influence of perfectionism and self-esteem on EA. Perhaps those environmental or internal personal traits, which are the so-called “trait factors,” affect EA and increase a tendency to avoid unpleasant internal states. In this study, stressor and psychological stress response, which can be called “state factors,” were also factors influencing EA, perhaps because stressor and psychological stress response are sometimes stimuli that are too negative to accept, and perhaps because psychological inflexibility develops from human suffering [18]. However, to avoid internal states would predict subsequent psychological distress, or pain and distress, and pain predicts subsequent avoidance [28]. It is thought that our results would add to the past research in support of the findings of the role of EA in the *process* of psychological well-being, and that they would provide some evidence about the mechanism involved in the maintenance of psychological distress among adolescents.

Some limitations of the present study suggest the necessity of further research on adolescent psychological well-being. First, we investigated the reciprocal relations between EA, stressor, and psychological stress response based on a self-reported questionnaire, which has inherent potential for subjective response bias. Further research may need to include methods to increase objectivity, such as teacher or parent reports or questionnaires. Second, we used short-term intervals between the measurements to find the process by which EA and psychological stress response influence each other. Though it may be beneficial to understand the temporal relations between these factors, some cross-lagged effects found in this study were relatively weak. Too-short intervals may lead to the conclusion that no causal effects exist [29]. We think one-week intervals may have been suitable for investigating the temporal relation of EA and psychological stress response, but perhaps because of the short timeframe between the lags we could not show strong prospective relations. To understand the causal relations of EA and stress in adolescents, a longitudinal study of more than one year will be needed.

In summary, EA is related to subsequent psychological stress response. Moreover, stressor and psychological stress response that are affected by prior EA are also influenced by subsequent EA. Therefore, in this study we found that adolescent EA, stressor, and stress responses were reciprocally related in the short term. Future research would benefit from more detailed investigations of psychological inflexibility.
